# From use to abuse: psychological, neurobiological, and spiritual pathways in relational harm and recovery

**DOI:** 10.3389/fnbeh.2026.1805594

**Published:** 2026-05-08

**Authors:** Shilpa Bhardwaj, Arif Ali, Fayaz Ahmad Paul

**Affiliations:** 1 Department of Neurochemistry, Institute of Human Behavior and Allied Sciences (IHBAS), Delhi, India; 2 Department of Psychiatric Social Work, Institute of Human Behavior and Allied Sciences (IHBAS), Delhi, India; 3 Department of Social Work, Rajagiri College of Social Sciences, Kochi, India

**Keywords:** abuse, neuroscience, pathways, psychological, recovery, spirituality, use

## Abstract

This viewpoint examines the distinction between relational “use” and “abuse” through psychological, neurobiological, relational, and spiritual lenses. It conceptualizes use as a form of mutual and ethical interdependence, whereas abuse is understood as a persistent pattern of control that undermines an individual’s autonomy and self-worth. The paper highlights the profound impact of chronic emotional abuse on both mind and body, including dysregulation of stress-response systems, alterations in brain functioning, increased inflammation, and accelerated biological aging. It further emphasizes that the experience and impact of abuse vary across gender, age, and sociocultural contexts, often remaining unrecognized due to prevailing cultural norms. Additionally, the paper explores the role of spirituality in recovery, suggesting that it can facilitate meaning-making, enhance emotional regulation, and foster resilience, while acknowledging that its effectiveness is not universal. Ultimately, the paper positions relational abuse as a significant public health concern that requires nuanced, interdisciplinary, and culturally sensitive responses.

## Foundations of healthy relationships and the shift toward harm

Relationships are fundamental to human existence and well-being. Throughout life, interpersonal relationships offer emotional control, attachment security, identity, social significance, and practical support. Healthy relationships are defined by reciprocity, mutual recognition, and adaptive interdependence. They let people depend on each other while keeping autonomy and dignity. Emotional interactions in such partnerships provide solace and validation, especially during vulnerability or stress (Feeney and Collins, 2015). However, when dynamics shift from reciprocity to coercion, domination, and degradation, relationships can cause significant psychological harm. Understanding this shift from use to abuse marks not just a linguistic difference but reflects fundamentally distinct relational frameworks, regulatory mechanisms, and biopsychosocial consequences.

## Conceptual distinction between use and abuse

The difference between “use” and “abuse” in relationships is not just semantic. It reflects fundamentally different relational structures, power dynamics, and biopsychosocial processes (Postmus et al., 2016). Clarifying this difference is important for clinical assessment and research. Relational distress may result from either temporary imbalances or entrenched harmful patterns. These forms of distress need different responses. To improve conceptual clarity, this framework defines the difference between use and abuse along five dimensions: intentionality, duration, power dynamics, impact on autonomy and dignity, and capacity for repair. “Use” describes negotiated interdependence, in which people rely on one another for emotional, psychological, or material needs within ethical boundaries (Walker, 1979). Reciprocity may shift temporarily due to life’s challenges, such as illness, distress, financial problems, or transitions (Rakovec-Felser, 2014). Such shifts are not inherently harmful. They occur within a system of mutual recognition, respect for autonomy, and a commitment to the relationship. Operationally, use is situational, arising from genuine need rather than coercion. Dependence is temporary, with an expectation of eventual balance. Agency is preserved so individuals retain voice, choice, and dignity (Mirpour and Shafiei Rezvani Nejad, 2025).

Importantly, relationships characterized by use maintain a capacity for repair, with communication, boundary-setting, and emotional attunement facilitating the restoration of equilibrium.

In contrast, “abuse” constitutes a distinct relational phenomenon marked by systematic patterns of coercive control, intimidation, humiliation, and psychological manipulation that function to erode an individual’s autonomy and sense of self (O’Leary and Maiuro, 2001; Johnson, 2008). Unlike use, abuse is defined by coercive intentionality, where behaviors are enacted to dominate, exploit, or control the other person. It is typically chronic and self-reinforcing, escalating over time rather than resolving, and is embedded within asymmetrical power dynamics that privilege one individual at the expense of the other. The defining feature of abuse is not merely relational distress, since distress may occur in non-abusive relationships, but the systematic degradation of dignity, agency, and psychological integrity. Individuals subjected to abuse are often treated instrumentally, as objects for emotional regulation, behavioral compliance, or maintenance of the perpetrator’s power (Johnson, 2008). Positioning relationships along a continuum from healthy to abusive further clarifies this distinction.

Healthy relationships are characterized by mutual respect, equality, safety, and emotional security, enabling individuals to experience stability, trust, and psychological well-being (Murray et al., 2020; Canevello and Crocker, 2010). Unhealthy relationships, which occupy an intermediate position, may involve poor communication, recurring conflict, or emotional dysregulation, yet still retain the potential for repair and do not fundamentally undermine autonomy (Umberson and Montez, 2010). Abusive relationships, however, are anchored in power and control, often generating persistent feelings of fear, insecurity, hypervigilance, and emotional exhaustion. Individuals may describe experiences of “walking on eggshells,” reflecting an environment of unpredictability and psychological threat (Wildman et al., 2023). Importantly, abusive behavior is frequently misunderstood as overt or uncontrolled aggression, whereas empirical evidence suggests that it is often calculated, selective, and context-dependent. Perpetrators may present as socially competent or even charming in public settings while directing abusive behaviors toward specific individuals in private contexts. This selective enactment enables concealment, denial, and minimization of abuse, often contributing to victim self-doubt and gaslighting (Smith, 2024). Such patterns highlight the importance of assessing abuse not solely through isolated incidents but through consistent behavioral patterns and underlying power structures.

The dynamics of abuse are further illuminated by the cycle of violence, which conceptualizes abuse as a repetitive and predictable pattern comprising phases of tension building, acute incident, reconciliation (or “honeymoon”), and temporary calm (Walker, 1979, 1981). This cyclical pattern reinforces emotional dependency and contributes to the development of learned helplessness, wherein individuals perceive limited control over their circumstances despite ongoing harm. Over time, repeated exposure to such cycles may result in hypervigilance, emotional numbing, disrupted interpersonal functioning, and difficulties in leaving abusive relationships (Walker, 1979; Herman et al., 2016). Intermittent reinforcement, particularly during reconciliation phases characterized by apology, affection, or promises of change, further strengthens attachment bonds and complicates disengagement. Theoretical perspectives provide additional insight into the structural and interpersonal mechanisms underlying abuse. Feminist theory situates intimate partner violence within broader systems of gender inequality and patriarchal power, conceptualizing abuse as an expression of dominance and control reinforced by sociocultural norms (Dobash and Dobash, 1979). Conflict theory emphasizes the role of competing interests and power struggles within relationships, in which violence may emerge as a maladaptive strategy for resolving disputes (Loseke et al., 2005).

Social learning theory, as articulated by Bandura (1977), highlights the roles of observational learning, reinforcement, and modeling in the development of abusive behaviors, particularly within family contexts where aggression may be normalized or rewarded. Together, these frameworks underscore that abuse is not solely an individual pathology but is shaped by relational, developmental, and sociocultural influences (Dworkin and Weaver, 2021). From a clinical and ethical perspective, distinguishing between use and abuse is critical for guiding intervention. While relational use may benefit from therapeutic approaches that emphasize communication, emotional regulation, and boundary negotiation, abuse necessitates protective, trauma-informed, and often multi-systemic responses aimed at ensuring safety and restoring autonomy (Trickett et al., 2011; Dworkin and Weaver, 2021). Recognizing the operational criteria outlined above enables practitioners to differentiate reparable relational distress from violations of human dignity, thereby facilitating more precise, compassionate, and effective responses. Moreover, integrating psychological and neurobiological perspectives highlights how trauma, attachment insecurity, and stress dysregulation both contribute to and result from abusive dynamics (Herman et al., 2016; González-Acosta et al., 2021).

Table 1 presents a comparative framework that distinguishes “use” (negotiated interdependence) from “abuse” (coercive control) across key relational dimensions, including intentionality, duration, power dynamics, autonomy, emotional climate, and capacity for repair. It highlights that while “use” reflects adaptive, reciprocal, and context-dependent dependence within ethical boundaries, “abuse” is characterized by persistent patterns of domination, control, and erosion of individual autonomy.

**TABLE 1:** Conceptual distinction between “use” and “abuse” in relationships.

Dimension	Use (negotiated interdependence)	Abuse (coercive control)
Intentionality	Driven by genuine need, care, and mutual support	Driven by control, domination, or exploitation
Duration and pattern	Situational, temporary, and context-dependent	Chronic, repetitive, and escalating over time
Power dynamics	Relatively balanced, flexible, and reciprocal	Asymmetrical, rigid, and hierarchical
Autonomy and agency	Preserved; individuals retain voice, choice, and dignity	Undermined; autonomy is restricted or eroded
Emotional climate	Safety, trust, and mutual respect	Fear, unpredictability, hypervigilance
Impact on self	Supports growth, resilience, and well-being	Leads to shame, confusion, and emotional exhaustion
Capacity for repair	High; conflicts resolved through communication and boundary-setting	Low; patterns persist despite apologies or “honeymoon” phases
Dependence	Temporary and adaptive	Enforced, prolonged, and controlling
Behavioral pattern	Open, transparent, and accountable	Often hidden, selective, and manipulative (e.g., gaslighting)
Outcome	Relationship stability and mutual development	Psychological harm and erosion of identity

**FIGURE 1:**
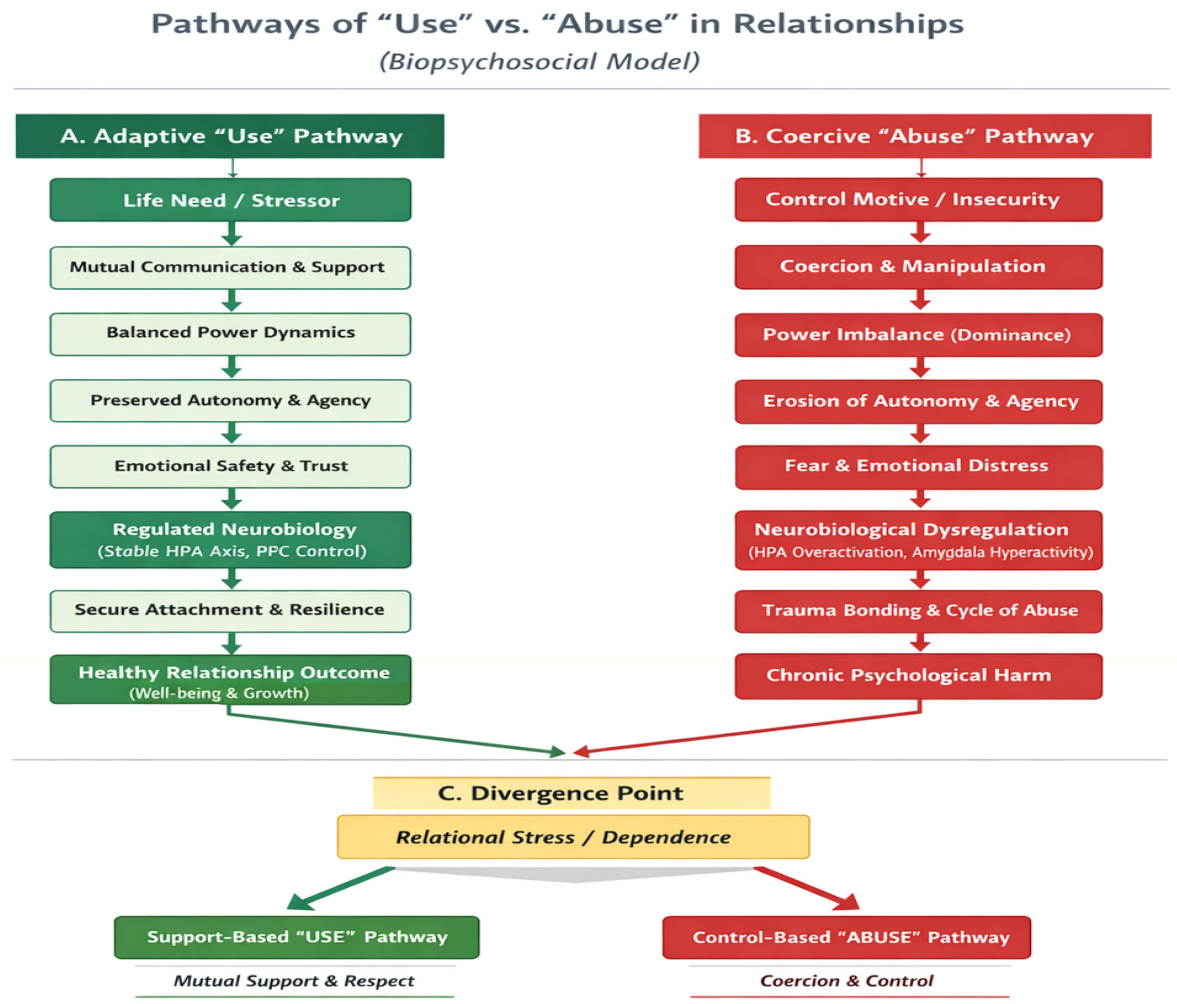
Pathways of “use” and “abuse” in relationships: a biopsychosocial model.

The Figure 1 illustrates the divergent pathways through which relational dependence may evolve into either adaptive “use” (negotiated interdependence) or maladaptive “abuse” (coercive control). While the use pathway is characterized by mutual support, balanced power, preserved autonomy, and regulated neurobiological functioning, the abuse pathway reflects coercion, power asymmetry, emotional distress, and neurobiological dysregulation, leading to trauma and psychological harm.

## Emotional abuse across gender, life stages, and intersectional contexts

Emotional abuse is widely recognized within psychological literature as a central component of intimate partner violence (IPV), encompassing behaviors such as verbal aggression, humiliation, ridicule, coercive control, social isolation, and symbolic intimidation, including threats to valued relationships or property (Karakurt and Silver, 2013; Jung et al., 2019). While gender and age are critical determinants shaping the experience and expression of emotional abuse, a more comprehensive understanding requires an intersectional perspective that considers how these factors interact with broader socio-cultural and structural conditions. Historically, IPV research has predominantly focused on women of reproductive age, often overlooking other affected populations, including men, older adults, and individuals from diverse socio-economic and cultural backgrounds. Emerging evidence suggests that men also experience emotional abuse at comparable rates, particularly in forms involving verbal aggression, control, and psychological manipulation, although underreporting remains a significant concern due to stigma and gender norms (Karakurt and Silver, 2013; Hosain, 2025). At the same time, women may experience more severe and cumulative forms of abuse linked to structural inequalities, including economic dependency and gendered power imbalances. Age further shapes the nature and impact of emotional abuse. Younger individuals may encounter abuse in the context of identity formation and relational dependency.

In contrast, older adults, particularly women, may experience abuse through neglect, increased control, and financial exploitation, often compounded by declining health, caregiving reliance, and social isolation (Dokkedahl et al., 2022). These vulnerabilities highlight the importance of situating emotional abuse within a life-course framework. An intersectional lens reveals that experiences of emotional abuse are not uniform but are shaped by overlapping social identities and structural determinants, including socio-economic status, cultural norms, and social marginalization (Adepoju et al., 2025). Individuals from lower socio-economic backgrounds may face heightened dependency and limited access to support systems, increasing vulnerability to sustained abuse (Gueta, 2020). Cultural contexts may influence the normalization, reporting, and interpretation of abusive behaviors, with certain controlling practices being minimized or justified within specific social frameworks (Childress et al., 2023). Additionally, stigma and lack of institutional support may further silence victims across genders and age groups (Dabagh Fekri et al., 2025). Perpetrators often employ a range of tactics, including intimidation, humiliation, economic control, and the manipulation of children to maintain dominance and undermine autonomy (Johnson, 2008; Sullivan et al., 2022). Emotional abuse may occur independently or alongside physical or sexual violence, collectively functioning to erode agency and reinforce power asymmetries (Pence and Paymar, 1993).

## Psychological impact of chronic emotional abuse

A persistent experience of exploitation within intimate relationships, or the gradual internalization of utilitarian relational dynamics, can profoundly shape personality development and psychosocial functioning over time. Unlike episodic physical aggression, emotional abuse is often chronic, subtle, and embedded within everyday interactions, making it less visible yet deeply pervasive in its psychological effects (Dye, 2019; Rakovec-Felser, 2014). Through repeated patterns of manipulation, control, criticism, and invalidation, emotional abuse progressively alters an individual’s self-concept, interpersonal expectations, and emotional regulation capacities. One of the most significant consequences of chronic emotional abuse is the erosion of self-esteem and identity. Individuals subjected to ongoing psychological harm often internalize negative messages conveyed by the perpetrator, leading to distorted self-perceptions characterized by inadequacy, guilt, and self-blame (Dye, 2019). Over time, this internalization can result in cognitive distortions, such as believing oneself to be responsible for the abuse or deserving of mistreatment. These maladaptive beliefs are further reinforced by cycles of intermittent validation and devaluation, which create confusion and undermine the individual’s capacity for accurate self-appraisal. Chronic emotional abuse also disrupts emotional regulation processes (Waseem and Firdous, 2025).

Persistent exposure to unpredictable and invalidating environments can lead to heightened emotional reactivity, difficulty identifying and expressing emotions, and reliance on maladaptive coping strategies (Dye, 2019). Individuals may adopt coping mechanisms such as emotional withdrawal, avoidance, dissociation, or hypervigilance as protective responses to ongoing psychological threat. While these strategies may offer short-term relief, they often impair long-term functioning by limiting emotional processing and reinforcing relational disengagement. Furthermore, prolonged exposure to abusive dynamics can result in learned helplessness, wherein individuals perceive a diminished sense of control over their circumstances and reduced capacity to initiate change (Nuvvula, 2016). This state is frequently accompanied by feelings of hopelessness, passivity, and dependency, which may contribute to the maintenance of abusive relationships. Interpersonally, individuals may develop mistrust, fear of intimacy, or maladaptive relational schemas, which can affect their ability to form and sustain healthy relationships in the future (Palker-Corell and Marcus, 2004). Importantly, these psychological impacts are cumulative and may persist even after the abusive context has ended.

## Developmental and relational escalation of conflict

The influence of relational experiences is not static but evolves across the life course, as expectations, roles, and meanings attached to relationships shift with age and developmental stage. Although the association between relationship status and mental well-being has been widely examined, age is often treated as a mere control variable rather than as a central developmental factor shaping relational dynamics (Hsu and Barrett, 2020; Bulloch et al., 2017). In reality, each life stage is characterized by distinct relational goals, such as attachment formation in early adulthood, stability and caregiving in midlife, and dependency or support needs in later life, which significantly influence how individuals perceive, tolerate, and respond to relational conflict and harm (Wadsworth, 2016). From a developmental perspective, the transition from perceived “use” to emotional abuse is closely linked to dysfunctional conflict resolution processes and deficits in emotional regulation. In healthy relationships, conflict is typically managed through communication, negotiation, and repair. However, in contexts marked by attachment insecurity, poor communication skills, or unmet emotional needs, conflict may escalate into patterns of psychological aggression. Theories of family conflict and escalation suggest that unresolved disagreements can progressively intensify, moving from verbal disagreements to hostility, coercion, and emotional abuse when adaptive coping mechanisms are absent (Holtzworth-Munroe et al., 2000; Whitaker et al., 2007). Importantly, developmental vulnerabilities such as limited emotional awareness in younger individuals or increased dependency in older adults may heighten susceptibility to such escalation. Over time, repeated cycles of unresolved conflict can normalize maladaptive interaction patterns, reinforcing power imbalances and emotional harm.

## Neurobiological effects of prolonged abuse

Prolonged exposure to emotional abuse constitutes a chronic stressor that persistently activates the hypothalamic–pituitary–adrenal (HPA) axis, resulting in dysregulated cortisol secretion–the primary glucocorticoid involved in stress regulation (Herman et al., 2016). While acute activation of the stress response is adaptive and supports survival through energy mobilization and “fight-or-flight” mechanisms, sustained activation leads to allostatic load, reflecting cumulative physiological strain across multiple regulatory systems (McEwen, 2007; Arnaldo et al., 2022). Over time, this dysregulation impairs the body’s ability to return to homeostasis, increasing vulnerability to a wide range of psychiatric conditions, including depression, anxiety disorders, and post-traumatic stress disorder, as well as somatic illnesses such as cardiovascular disease and metabolic dysfunction (Shchaslyvyi et al., 2024).

Advances in neuroimaging and neurobiological research provide compelling evidence that chronic emotional abuse results in structural and functional alterations in key brain regions. The amygdala, central to threat detection and fear processing, often exhibits hyperactivation, contributing to persistent hypervigilance and exaggerated stress responses. Concurrently, the hippocampus, implicated in memory consolidation and contextual processing, may show reduced volume and impaired functioning, affect the integration of emotional experiences, and increase susceptibility to intrusive memories and maladaptive learning patterns (Teicher et al., 2016; González-Acosta et al., 2021). The prefrontal cortex, particularly the medial and dorsolateral regions, plays a critical role in executive functioning, impulse control, and emotional regulation. Chronic stress exposure has been associated with diminished prefrontal regulatory capacity, resulting in impaired decision-making, reduced cognitive flexibility, and difficulties in inhibiting maladaptive responses. This imbalance between heightened limbic reactivity and weakened cortical control mechanisms underlies many of the behavioral and emotional patterns observed in individuals exposed to prolonged relational abuse, including difficulty disengaging from harmful relationships despite conscious awareness of risk (Wong et al., 2014).

At a systems level, prolonged abuse disrupts neural connectivity, particularly between the prefrontal cortex and limbic structures, leading to inefficient top-down regulation of emotional responses. Functional neuroimaging studies also indicate alterations in the default mode network and salience network, which are involved in self-referential processing and threat detection, respectively. These disruptions may contribute to persistent negative self-appraisals, rumination, and heightened sensitivity to perceived interpersonal threats. Importantly, these neurobiological adaptations are not merely correlational but reflect experience-dependent plasticity, wherein repeated exposure to stress and coercion shapes neural architecture over time. While such adaptations may initially serve protective functions enhancing vigilance and responsiveness to threat, they become maladaptive when chronically activated, reinforcing cycles of anxiety, emotional dysregulation, and relational dependency. This neurobiological embedding of trauma helps explain why individuals subjected to prolonged emotional abuse often experience enduring psychological distress and face challenges in achieving recovery even after the cessation of overt abuse (Herman et al., 2016; Teicher et al., 2016).

Table 2 summarizes the neurobiological differences between adaptive relational functioning (“use”) and abusive relational dynamics. It outlines the involvement of key systems, including the stress response system (HPA axis), the amygdala, the prefrontal cortex, the hippocampus, the attachment system, and the rewards pathways. While healthy relational contexts are associated with regulated stress responses and stable emotional processing, abusive environments are linked to chronic stress activation, fear-related hyperarousal, impaired regulation, trauma-related memory disruptions, and maladaptive attachment patterns.

**TABLE 2:** Neurobiological correlates of abuse vs. adaptive use.

System	In healthy/use context	In abuse context
HPA axis (stress response)	Regulated, adaptive stress response	Chronic activation, toxic stress
Amygdala	Normal threat detection	Hyperactivation → fear, hypervigilance
Prefrontal cortex	Intact regulation, decision-making	Reduced control, impaired judgment
Hippocampus	Stable memory processing	Trauma-related memory disturbances
Attachment system	Secure attachment patterns	Insecure/disorganized attachment
Rewards system (dopamine)	Stable bonding and reinforcement	Intermittent reinforcement (trauma bonding)

## Hormonal and biological embedding of trauma

Beyond neural alterations, chronic emotional abuse exerts profound effects on endocrine and physiological systems, contributing to the biological embedding of trauma. Hormonal dysregulation plays a central role in shaping both the emotional and behavioral dimensions of abusive relationships. Oxytocin, typically associated with bonding, trust, and social affiliation, may paradoxically reinforce attachment in contexts of fear and unpredictability. Under conditions of intermittent reinforcement, where episodes of abuse are followed by affection or reconciliation, oxytocin release may strengthen emotional bonds, contributing to what is often described as trauma bonding (Cardoner et al., 2024). Dopaminergic pathways further complicate this dynamic. Intermittent positive reinforcement activates the brain’s rewards system, producing transient feelings of relief, pleasure, or hope. This cyclical activation mirrors reinforcement patterns observed in addiction, where unpredictable rewards strengthen behavioral persistence. As a result, individuals may become emotionally conditioned to remain within abusive relationships, despite ongoing harm, due to the powerful neurochemical associations between intermittent affection and rewards.

Cortisol dysregulation remains a central feature of chronic stress exposure. Interestingly, research indicates variability in cortisol responses, with some individuals exhibiting hypercortisolism and others showing blunted or hypocortisol responses, depending on the chronicity and severity of trauma (Mbiydzenyuy and Qulu, 2024). This variability reflects adaptive recalibration of the stress system but is associated with increased risk for mood disorders, immune dysfunction, and impaired stress resilience. The use of biomarkers, including salivary, plasma, and hair cortisol, has enabled more precise assessment of long-term stress exposure and its physiological correlates. In addition to endocrine disruption, chronic relational stress is increasingly linked to systemic inflammation. Elevated levels of inflammatory markers such as C-reactive protein (CRP), interleukin-6 (IL-6), and tumor necrosis factor-alpha (TNF-α) have been observed in individuals exposed to prolonged interpersonal trauma (Slevin and Tero-Vescan, 2025).

Persistent inflammation contributes to the development of a range of chronic health conditions, including cardiovascular disease, autoimmune disorders, and neurodegenerative processes. This inflammatory response reflects the body’s prolonged activation of defense mechanisms in the absence of physical injury, underscoring the physiological reality of emotional abuse. At the cellular level, emerging evidence indicates that chronic stress shortens telomere length, a biomarker of cellular aging. Telomeres, which protect chromosomal integrity, progressively shorten with repeated cell division and exposure to stress (Humphreys et al., 2012). Studies have demonstrated associations between intimate partner violence and accelerated telomere shortening, suggesting that chronic emotional abuse may contribute to premature biological aging (Slevin and Tero-Vescan, 2025; Chan et al., 2023). Collectively, these hormonal and biological processes illustrate how emotional abuse extends beyond psychological harm to produce measurable, long-term physiological consequences. The integration of neuroendocrine, immune, and cellular findings reinforces the conceptualization of relational trauma as a whole-body condition, necessitating interdisciplinary approaches to assessment, intervention, and recovery.

## Existential disruption and spirituality as a framework for trauma recovery

Human beings often experience an existential tension between “use,” characterized by ethical, constructive engagement with the self and others, and “abuse,” which reflects exploitative and dehumanizing relational patterns. The collapse of this balance can lead to profound disruptions in meaning, dignity, and psychological stability (Staton-Tindall et al., 2013). Within this context, spirituality has increasingly been recognized as a potential pathway for healing and recovery. However, its role is complex and multifaceted; spirituality is not a monolithic construct, and different spiritual practices may exert distinct psychological and neurobiological effects (de Brito Sena et al., 2021; Lucchetti and Lucchetti, 2014). A nuanced understanding of these differentiated pathways is essential for evaluating their relevance and effectiveness in trauma recovery. One of the most extensively studied spiritual pathways is meditation and mindfulness-based practice, which primarily operates through attentional regulation and physiological modulation of stress responses (Schuman-Olivier et al., 2020; Keng et al., 2011). These practices cultivate non-judgmental awareness of present-moment experiences, enabling individuals to observe distressing thoughts and emotions without becoming overwhelmed. For individuals exposed to chronic emotional abuse, who often experience hypervigilance, intrusive thoughts, and emotional dysregulation, such practices can be particularly beneficial. Neurobiologically, meditation has been associated with reduced hypothalamic–pituitary–adrenal (HPA) axis activation, decreased cortisol levels, and enhanced parasympathetic nervous system activity, thereby facilitating a shift from states of chronic arousal to physiological calm (Howard et al., 2023). Functional neuroimaging studies further indicate increased connectivity between the prefrontal cortex and limbic regions, supporting emotional regulation, impulse control, and cognitive flexibility (Herman et al., 2016). Over time, sustained meditative practice has been linked to structural brain changes, including increased cortical thickness in regions associated with attention and self-awareness, suggesting its potential role in long-term neurobiological recovery.

## Differential mechanisms of spiritual coping: prayer and altruistic engagement

In contrast, prayer and devotional practices engage mechanisms that are more relational and attachment-oriented. These practices often involve communication with or surrender to a perceived higher power, providing individuals with a sense of emotional containment, guidance, and existential security. For trauma survivors, particularly those whose interpersonal trust has been compromised by abusive relationships, prayer may serve as an alternative or supplementary attachment system, fostering feelings of being supported and protected. This relational dimension can mitigate loneliness, enhance coping capacity, and facilitate emotional expression. However, the effectiveness of prayer is highly contingent on the nature of one’s spiritual beliefs. Positive religious coping, characterized by beliefs in a benevolent, forgiving, and supportive higher power, is associated with improved mental health outcomes, including reduced anxiety and depressive symptoms (Xu, 2016; Philip et al., 2025). Conversely, negative religious coping, which involves beliefs in divine punishment, abandonment, or moral condemnation, has been linked to increased psychological distress and maladaptive coping patterns. Thus, while prayer can be a powerful resource, its impact is mediated by cognitive and theological interpretations.

A third important pathway is altruistic engagement or selfless service (seva), which operates through mechanisms of meaning-making, identity reconstruction, and prosocial orientation. Trauma, particularly in the context of relational abuse, often disrupts an individual’s sense of purpose and moral coherence. Engaging in acts of service redirects attention from self-focused distress toward collective well-being, fostering empathy, connectedness, and a renewed sense of agency. This outward orientation can counteract the helplessness and passivity that frequently accompany chronic abuse. Empirical studies suggest that prosocial behavior is associated with enhanced psychological well-being, reduced depressive symptoms, and improved social integration (Anderson et al., 2012). In culturally embedded contexts, such as South Asia, seva is not only a social act but also a spiritual discipline that reinforces values of interconnectedness, humility, and ethical responsibility. By reframing one’s role from victimhood to contribution, individuals may reconstruct their identities in more empowering ways.

## Meaning-making, neurobiological correlates, and the limits of spiritual coping

Across these diverse pathways, spirituality contributes to meaning-making, a central process in trauma recovery (Wortmann et al., 2011). Meaning-making involves the reinterpretation of adverse experiences within broader existential or moral frameworks, enabling individuals to integrate trauma into coherent life narratives. This process has been shown to enhance resilience, promote psychological growth, and reduce the long-term impact of trauma (Park, 2022). Practices such as gratitude and forgiveness play a crucial role in this context. Gratitude shifts attention from loss and deprivation to appreciation and possibility, thereby enhancing emotional regulation and well-being. Forgiveness, while complex and context-dependent, can help release persistent anger and resentment, reducing psychological burden. Importantly, these processes do not imply condoning abuse but rather reflect adaptive strategies for emotional healing (Breen et al., 2010). Emerging evidence also highlights the neurobiological correlates of spiritual practices. Meditation, prayer, and communal rituals have been associated with modulation of stress-response systems, including reduced cortisol secretion and increased parasympathetic activity (Howard et al., 2023). Additionally, these practices may enhance the release of neurochemicals such as oxytocin and β-endorphins, which promote social bonding, emotional safety, and stress reduction. Increased levels of brain-derived neurotrophic factor (BDNF) have also been observed, suggesting potential roles in synaptic plasticity and neural repair (Philip et al., 2025). Functional neuroimaging studies indicate reduced hyperactivity in the default mode network, which is associated with rumination and self-referential thinking, and improved regulation between prefrontal and limbic systems, facilitating emotional stability and insight (Herman et al., 2016). These findings collectively support the view that spirituality can contribute not only to psychological but also to neurobiological healing.

Despite these potential benefits, it is essential to recognize the limitations and risks associated with spiritual coping. Spirituality does not provide universal protection, and in some cases, it may inadvertently perpetuate harm. Negative religious coping, as noted earlier, is associated with increased levels of depression, anxiety, and even suicidality (Xu, 2016; Philip et al., 2025). In certain sociocultural contexts, rigid or fundamentalist interpretations of spiritual teachings may discourage help-seeking, promote endurance of suffering, or legitimize abusive dynamics. For example, beliefs that emphasize submission, sacrifice, or divine testing may lead individuals to remain in harmful relationships, delaying intervention and increasing risk (Laurin et al., 2012; Martel et al., 2021). Furthermore, overreliance on spiritual practices in the absence of professional support may limit access to evidence-based psychological interventions.

## Limitations of existing evidence and future research directions

While the manuscript draws on a growing body of interdisciplinary research to elucidate the psychological and neurobiological impacts of emotional abuse, it is important to acknowledge the limitations inherent in the existing evidence base critically. A significant proportion of studies in this area rely on cross-sectional designs, which restrict the ability to establish causal relationships between exposure to abuse and observed psychological or biological outcomes (Tura et al., 2026). Longitudinal data, though emerging, remain comparatively limited, thereby constraining our understanding of temporal dynamics and long-term trajectories of recovery or deterioration. Additionally, many neurobiological and biomarker studies are conducted on small or highly specific samples, often drawn from clinical or high-risk populations, which may not adequately represent the broader population experiencing emotional abuse. Another important limitation relates to cultural and contextual bias. Much of the literature originates from Western contexts, where conceptualizations of abuse, relational norms, and coping mechanisms may differ significantly from those in non-Western settings such as South Asia (Rashid et al., 2025).

This raises concerns about the generalizability and cultural validity of the findings, particularly when applied to diverse populations with varying socio-cultural frameworks, family structures, and belief systems. Furthermore, there is considerable heterogeneity in the operationalization and measurement of emotional abuse, with studies employing differing definitions, scales, and thresholds, thereby complicating direct comparison and synthesis of findings across research (Wolke et al., 2025). Methodologically, variability in statistical approaches, including inconsistent reporting of effect sizes, confounding variables, and control measures, further limits the robustness of conclusions. Potential biases such as self-report bias, recall bias, and underreporting, particularly in sensitive areas like intimate partner violence, also warrant consideration. Addressing these limitations in future research through larger, longitudinal, and culturally grounded studies using standardized measures will be essential to strengthen the evidence base and enhance its applicability across diverse clinical and policy contexts.

## Implications

The distinction between relational “use” and “abuse” offers a critical conceptual lens for understanding how interpersonal dynamics can either sustain well-being or contribute to profound psychological and biological harm. While use reflects negotiated interdependence grounded in dignity, respect, and mutual care, abuse represents a systematic violation of autonomy, marked by coercion, control, and the erosion of selfhood. Recognizing this distinction is essential not only for theoretical clarity but also for guiding meaningful intervention across clinical, policy, and research domains. From a clinical perspective, there is a pressing need to move beyond a narrow focus on physical violence and systematically assess emotional and psychological abuse within routine practice. Mental health professionals should incorporate structured screening tools and trauma-informed frameworks to identify patterns of coercive control, even when overt violence is absent. Interventions must address both psychological and biological sequelae, integrating therapies such as trauma-focused cognitive behavioral therapy, emotion regulation approaches, and somatic interventions to target stress dysregulation. Given evidence of neurobiological embedding, clinicians should also consider the roles of chronic stress, sleep disturbances, and somatic symptoms in treatment planning. The integration of spirituality, where culturally appropriate, can support meaning-making and resilience; however, it must be applied critically to avoid reinforcing harmful endurance or self-blame.

At the level of policy and systems, emotional abuse must be recognized as a serious public health concern with long-term consequences. Legal and welfare frameworks often prioritize physical forms of violence, leading to the invisibility of psychological harm. Policymakers should expand definitions of intimate partner violence to explicitly include emotional abuse and ensure access to integrated services, including mental health care, legal aid, shelter, and social rehabilitation. In contexts such as urban India, where service delivery is fragmented, intersectoral coordination between healthcare, social welfare, and community organizations is essential. In the long term, recovery-oriented models that emphasize safety, stability, and reintegration should replace short-term crisis responses. From a research standpoint, the field would benefit from more rigorous and integrative methodologies. Longitudinal studies are particularly needed to establish causal pathways linking emotional abuse to psychological, neurobiological, and physical health outcomes. Future research should prioritize underrepresented populations, including men, older adults, and marginalized groups, to challenge existing gendered and age-restricted assumptions. Additionally, there is a need for interdisciplinary approaches that bridge psychology, neuroscience, and social sciences, as well as for the standardization of measures assessing emotional abuse and biological markers to enhance comparability and reproducibility. Finally, capacity building and public awareness are crucial. Training programmes for clinicians, social workers, law enforcement, and community stakeholders should emphasize the identification and management of non-physical forms of abuse. Public health campaigns must work to challenge societal norms that normalize emotional harm and silence victims. By fostering awareness and equipping systems with appropriate tools, it becomes possible to intervene earlier and more effectively. In conclusion, addressing relational abuse requires a holistic, trauma-informed, and culturally sensitive approach that bridges theory and practice. Integrating clinical insight, policy reform, and research advancement is essential to prevent harm, restore dignity, and promote sustainable recovery.

## Conclusion

The distinction between relational “use” and “abuse” is central to understanding how relationships can either support well-being or produce enduring harm. While use reflects adaptive, negotiated interdependence grounded in respect and autonomy, abuse represents a systematic erosion of dignity through coercion, control, and psychological harm. This viewpoint highlights that emotional abuse is not only a psychosocial issue but also a neurobiological and public health concern, with lasting effects on stress regulation, brain functioning, and physical health. Recognizing these multidimensional impacts is essential for timely identification and intervention. Recovery requires trauma-informed, culturally sensitive approaches that address both psychological and biological consequences, while also fostering meaning-making and resilience. Integrating clinical care, policy reform, and future research is critical to addressing the often-invisible nature of emotional abuse and promoting pathways toward healing, autonomy, and sustained well-being across diverse populations.

## Data Availability

The original contributions presented in this study are included in this article/supplementary material, further inquiries can be directed to the corresponding author.
